# Commensal Clostridia: leading players in the maintenance of gut homeostasis

**DOI:** 10.1186/1757-4749-5-23

**Published:** 2013-08-13

**Authors:** Loris R Lopetuso, Franco Scaldaferri, Valentina Petito, Antonio Gasbarrini

**Affiliations:** 1 Department of Internal Medicine, Gastroenterology Division, Catholic University of Rome, Policlinico “A. Gemelli” Hospital, Roma 00168, Italia; 2 Department of Pathology, Case Western Reserve University School of Medicine, Cleveland, OH 44106, USA

**Keywords:** Gut microbiota, Clostridia spp, Dysbiosis, Gut homeostasis

## Abstract

The gastrointestinal tract is a complex and dynamic network where an intricate and mutualistic symbiosis modulates the relationship between the host and the microbiota in order to establish and ensure gut homeostasis. Commensal Clostridia consist of gram-positive, rod-shaped bacteria in the phylum *Firmicutes* and make up a substantial part of the total bacteria in the gut microbiota. They start to colonize the intestine of breastfed infants during the first month of life and populate a specific region in the intestinal mucosa in close relationship with intestinal cells. This position allows them to participate as crucial factors in modulating physiologic, metabolic and immune processes in the gut during the entire lifespan, by interacting with the other resident microbe populations, but also by providing specific and essential functions. This review focus on what is currently known regarding the role of commensal Clostridia in the maintenance of overall gut function, as well as touch on their potential contribution in the unfavorable alteration of microbiota composition (dysbiosis) that has been implicated in several gastrointestinal disorders. Commensal Clostridia are strongly involved in the maintenance of overall gut function. This leads to important translational implications in regard to the prevention and treatment of dysbiosis, to drug efficacy and toxicity, and to the development of therapies that may modulate the composition of the microflora, capitalizing on the key role of commensal Clostridia, with the end goal of promoting gut health.

## Introduction

The gastrointestinal (GI) tract, man’s most widely exposed organ system to the external environment with a global surface of 200 m^2^, is a complex and dynamic network with interplay between various gut mucosal cells and their defense molecules, the immune system, food particles, and the resident microbiota. This ecosystem acts as a functional unit organized as a semipermeable multi-layer system that allows the absorption of nutrients and macromolecules required for human metabolic processes and, on the other hand, protects the individual from potentially invasive microorganisms [1,2]. These basic functions are carried out in a dynamic environment inhabited by 1kg of commensal microbes that include more than 3mln of genes [3,4]. They belong to the three domains of life, *Bacteria, Archaea* and *Eukarya*[5-7], as well as to viral particles [8,9]. Recent advances in culture-independent molecular techniques, by the analysis of phylogenetic arrays, next generation 16S rRNA sequencing and metagenome sequencing derived from human mucosal biopsies, luminal contents and feces, have shown that four major microbial phyla, (*Firmicutes, Bacteroides, Proteobacteria* and *Actinobacteria*), represent 98% of the intestinal microbiota and fall into three main groups of strict extremophile anaerobes: *Bacteroides, Clostridium* cluster *XIVa* (also known as the Clostridium Coccoides group), and *Clostridium* cluster *IV* (also known as the Clostridium leptum group) [5,6,10-17].

An intricate and mutualistic symbiosis modulates the relationship between the host and the gut microbiota [11,18,19]. This relationship is constantly challenged with several factors such as rapid turnover of the intestinal epithelium and overlaying mucus, exposure to peristaltic activity, food molecules, gastric, pancreatic and biliary secretions, defense molecules, drugs, pH and redox potential variations, and exposure to transient bacteria from the oral cavity and esophagus, and can lead to the collapse of the microbial community structure [17]. On the other hand, resident microbes perform several useful functions, including maintaining barrier function, synthesis and metabolism of nutrients, drug and toxin metabolism, and behavioral conditioning [20]. Gut microbiota is also involved in the digestion of energy substrates, production of vitamins and hormones [21], protection from pathogenic bacteria by consuming nutrients and producing molecules that inhibit their growth [22-24], production of nutrients for mucosal cells [25-27], augmenting total and pathogen-specific mucosal IgA levels upon infection [28,29], and in modulating immune system development and immunological tolerance [30].

Unfavorable alteration of microbiota composition, known as dysbiosis, has been implicated in chronic gut, and perhaps also systemic, immune disorders, such as in the pathogenesis of inflammatory bowel diseases (IBD), and other gastrointestinal disorders, including gastritis, peptic ulcer, irritable bowel syndrome (IBS) and even gastric and colon cancer [14,31-33].

In this scenario, gut commensal Clostridia consist of gram-positive, rod-shaped bacteria in the phylum *Firmicutes*. The *Clostridium* cluster *XIVa* and *IV*, known for many years as fusiform-shaped bacteria, make up a substantial part (10-40%) of the total bacteria in the gut microbiota [10,14,17]. As such, it is likely that Clostridia play a crucial role in gut homeostasis by interacting with the other resident microbe populations, but also by providing specific and essential functions. The purpose of this review is to explore and emphasize the critical role of intestinal commensal Clostridia in modulating normal gut homeostasis. Based on this, detailed mechanistic studies could improve the development of microbial therapies that may modulate the composition of the gut microflora, capitalizing on the key role of commensal Clostridia, with the end goal of promoting gut health.

## Gut barrier and commensal microbiota

The intestinal barrier is a functional unit, organized as a multi-layer system, in which it is possible to recognize two main parts: a superficial physical barrier, which prevents bacterial adhesion and regulates paracellular diffusion to the underlying host tissues, and a deeper functional barrier, which is able to discriminate commensal bacteria from pathogens and is responsible for immunological tolerance to commensal and immune response to pathogen microorganisms [20]. Everyday, thousands of compounds derived from food and microorganisms come in contact with the intestinal mucosa. This interaction requires a complex defense system that separates intestinal contents from the host tissues, regulates nutrient absorption, and allows tolerance between the resident bacterial flora and the mucosal immune system, while inhibiting translocation of infectious agents to the inner tissues. Commensal gut microbiota constitutes the anatomical barrier, along with the mucous layer and the intestinal epithelial monolayer. The deeper, inner layer consists of a complex network of immune cells organized in a specialized and compartmentalized system known as “gut-associated lymphoid tissue” or GALT. GALT represents both isolated and aggregated lymphoid follicles and is one of the largest lymphoid organs, containing up to 70% of the body’s total number of immunocytes and is involved in responding to pathogenic microorganisms, as well as providing immune tolerance to commensal bacteria. The ability of GALT to interact with the luminal antigens rests on specific mucosal immune cells (*i.e*., dendritic cells and M-cells), primarily localized to Peyer’s patches within the ileum that are intimately positioned at the mucosal-environmental interface and internalize microorganisms and macromolecules. These specialized immune cells have the ability to present antigen to naïve T-lymphocytes, which subsequently produce cytokines and activate mucosal immune responses, when needed. Thus, the mucosal immune system participates in the maintenance of gut microbial communities by directly monitoring the luminal environment through the constant sampling through M-cells that overlie lymphoid follicles and by dendritic cells that resides within the lamina propria. The interaction of these cellular components sustains the delicate equilibrium to maintain intestinal homeostasis, establishing a state of immunological tolerance towards antigens from food and commensal bacteria. Many factors can alter this balance, including alterations in the gut microflora, modifications of the mucus layer, and epithelial damage, leading to increased intestinal permeability and translocation of luminal contents to the underlying mucosa. Dysregulation of any of the aforementioned components have been implicated, not only in the pathogenesis of IBD, but in many other GI disorders, including infectious enterocolitis, IBS, small intestinal bowel overgrowth, celiac sprue, hepatic fibrosis, atopic manifestations and food intolerance [34-36].

The gut microbiota, which includes *Clostridium spp.*, is an essential actor in the aforementioned defense mechanisms and in the resistance to infection. It plays a crucial role, both by acting indirectly, for example in immune system development and modulating immunological tolerance [37], and also directly, by preventing potentially deleterious and pathogenic organisms from taking up residence. This phenomenon is known as colonization resistance [17].

## Commensal Clostridia in the GI tract

In healthy adults, each segment of the GI tract is populated with a distinct and stable community of microbes. *Escherichia, Clostidium, Lactobacillus, Bacteroides, Eubacterium, Peptococcus, Peptostreptococcus, Veillonella, Fusobacterium* and *Bifidobacterium* are the predominating microbial genera within the GI tract [38]. The distal ileum and colon harbor the highest amount of microbes with an estimated density of 1x10^12^ organisms per gram (dry weight) of feces [39]. Recent cultivation-independent 16S rRNA gene next generation sequencing techniques showed that in the colon, the most numerically predominant organisms belong to three groups of strict anaerobes: *Bacteroides, Clostridium* cluster *XIVa* and *Clostridium* cluster *IV*[10]. The *Clostridium* cluster *XIVa* includes species belonging to the *Clostridium, Eubacterium, Ruminococcus, Coprococcus, Dorea, Lachnospira, Roseburia* and *Butyrivibrio* genera. *Clostridium* cluster *IV* is composed by the *Clostridium, Eubacterium, Ruminococcus* and *Anaerofilum* genera [40]. Clostridia are gram-positive bacteria; they form endospores and therefore have an ecological advantage for survival under adverse conditions. Some Clostridia groups possesses pathogenic species, such as *Clostridium Perfrigens* and *Clostridium Tetani*, that are members of *Clostridium* cluster I, and *Clostridium Difficile*, a member of cluster XI. However, most of the Clostridia have a commensal relationship with the host.

Clostridia start to colonize the human intestine of breastfed infants during the first month of life [41]. Of note, infant-type microbiota is extremely plastic [42]. During birth, bacteria from the mother and the environment colonize the sterile GI tract of neonates. During the first few days of life, the bacterial flora is usually heterogeneous and independent of feeding habits. *Clostridium spp.* and other obligate anaerobes, such as *Bacteroides spp.*, are rarely isolated. Thereafter, the gradual consumption of oxygen by aerobic microorganisms decreases the oxidation-reduction potential, providing optimal conditions for a more diversified and stable bacterial flora, which also includes anaerobic bacteria [43]. The aging process challenges the stability of microbiota and can also affect the presence of *Clostridium spp.* In the elderly (>65 years), most available studies obtained with molecular methods showed a decrease in the number of strict anaerobes and an increase in the number of facultative anaerobes (streptococci, staphylococci, enterococci, enterobacteriaceae) [44-47]. These data suggest a decrease in the stability and in the diversity of the gut microbiota with advancing age [48]. However, Drago *et al*., studying 14 Italian centenarians, found a significant decrease in the total number of anaerobes compared with younger adults, caused by a lower count of bifidobacteria and bacteroides, whereas the *Clostridium* sensu stricto genus significantly increased [49], suggesting an indispensable and key role of Clostridia in modulating gut homeostasis during the entire lifespan.

Interestingly, Clostridia have been reported to colonize a specific part of the intestinal mucosa. In fact, it has been assessed that there is a spatial organization and diversity of microbes across the intestinal lumen [50]. The ascending colon of healthy mice is characterized by transverse folds, called the interfold region, that projects approximately 1mm into the lumen and is oriented perpendicular to the fecal stream [51]. Low-power magnification showed dense material between the transverse folds, while the central lumen (digesta region) displayed the presence of a less dense and less homogenous material, including food particles [50]. Higher magnification of these sections defined the material within the interfold region as fusiform-shaped bacteria (> 5-10 μm), whereas rod- and coccoid-shaped bacteria comprised the material of the digesta region. Laser capture microdissection revealed that the areas between the mucosal folds were populated by *Clostridium* cluster *XIVa* and *IV*, whereas the regions of the central lumen were enriched with Bacteroidaceae, Enterococcaceae and Lactobacillaceae [50]. Structural folds similar to the mouse interfold region are also found in the human intestine and are called plica lunaris and plica semilunaris in the small bowel and colon, respectively [50]. As such, it is possible that commensal Clostridia populate a specific region in the intestinal mucosa, establishing a close relationship with gut cells in order to perform critical physiological functions in a cooperative manner.

## Commensal Clostridia and gut homeostasis: the metabolic point of view

The microbiome, the whole genome of the gut flora that vastly exceeds the human genome [52], codes several biochemical pathways that are crucial for human life. These include the biosynthesis of the essential nutrient vitamins K and B12, the biotransformation of conjugated bile acids, the degradation of dietary oxalates, the carbohydrate and amino-acid methabolism, and the caloric extraction from otherwise indigestible polysaccharides. Furthermore, stimulation of TLR2 on colonic epithelial cells with bacterial antigens, such as LPS and peptidoglycan, initiates tight junction development leading to apical tightening and sealing and to an increased transepithelial electrical resistance [53]. Germ-free mouse models, compared to control animals, showed increased mucus accumulation in the cecum, water retention, extended epithelial cell cycles, and decreased peristalsis in the large intestine [54]. *Clostridium spp.*, due to their specific position in the mucosa, impact normal intestinal structure and physiology and are involved in the pathways of the aforementioned functions with continuous crosstalk with gut cells. In particular, commensal Clostridia play an important role in the metabolic welfare of colonocytes by releasing butyrate as an end-product of fermentation [55].

The short chain fatty acids (SCFAs), acetate, propionate and butyrate, non-gaseous fermentation end products of dietary components that are incompletely digested in the small intestine, provide a high proportion of the total energy gained from the diet in herbivores, especially ruminants [55]. In humans, SCFAs, in particular butyrate, are less involved in the host’s energy contribution, but instead seem to be the preferred energy source for colonocytes [56,57] and have an important influence on colonic health [58,59]. In fact, the colonic mucosa absorbs 95% of butyrate produced by butyrogenic bacteria, but concentrations in portal blood are usually undetectable as a result of rapid utilization. Thus, *Clostridium spp.* perform most of their metabolic functions through the release of butyrate that is essential as fuel for colonocytes. However, butyrate also influences gene expression through the hyperacetylation of chromatin through its action as a non-competitive inhibitor of histone deacetylases [60]. Moreover, butyrate inhibits the activation of the transcription factor, NF-kB, leading to decreased expression of proinflammatory cytokines and to a consequent anti-inflammatory effect [61,62]. Butyrate has also been implicated in protection against colitis and colorectal cancer [63-65]. In fact, butyrate has been shown to induce apoptosis in tumor cells *in vitro*[66] and, although colon carcinoma cells overexpress cyclooxygenase 2 and impart resistant to butyrate-induced apoptosis, it can act as a suppressing factor for pre-cancerous cells at an earlier stage of progression [60]. Depending on its concentration, butyrate is able to inhibit growth, but can also work as a trophic factor, inducing differentiation of human cells in tissue culture and preventing or ameliorating conditions, such as ulcerative colitis [58-60,65,67]. Harmful conditions that lead to a lack of energy supply to colonocytes, 70% of which is normally provided by butyrate, can be a causative factor in colitis and several reviews discuss its role in increasing the risks of both colorectal cancer and IBD [60,65,67]. In an animal model of colitis in which mice are orally administered dextran sodium sulfate (DSS), a T-cell independent colitis is induced that results in epithelial damage and acute inflammation, primarily driven by innate immune responses. Several potential mechanisms have been proposed to explain DSS-induced colitis. It could occur by inhibiting butyrate oxidation through sulfide toxicity, without affecting glucose metabolism, with a consequent inadequate energy supply to gut cells from butyrate [68,69].

Butyrate production is widely distributed among anaerobic bacteria belonging to the *Clostridial subphylum* and in particular, to the *Clostridial* clusters *XIVa* and *IV*, such as some potentially important butyrate producers related to *Roseburia* and *F. prausnitzii* that display Butyryl CoA:acetate CoA transferase activity [70]. However, very little information exists on the genetic predisposition and regulation of butyrate pathway enzymes in gut Clostridia. Most of the available data comes from industrial interest in solventogenic clostridia. More information could be helpful in developing prebiotic or probiotic strategies to take advantage of these essential metabolic roles of gut Clostridia.

## Commensal Clostridia and gut homeostasis: the brain-gut axis

Interestingly, a recent paper reported that gnotobiotic mice, associated with a mixture of 46 related *Clostridium spp.* from the Coccoides and Leptum groups, showed a drastic elevation of biologically active, free catecholamines, including Norepinephrine (NE) and Dopamine (DA), in the gut lumen compared to germ free mice [71]. Clostridia, enriched in β-glucuronidase activity, could be responsible for generating free NE and DA from the glucuronide-conjugated biologically inactive form that is normally released in the intestine. The gastrointestinal tract is densely innervated by noradrenergic and dopaminergic nerves, and their fibers are found in the gut mucosa, constituting part of the neuro-enteric system [72]. Catecholamines are utilized in the central and peripheral nervous systems, which regulate various types of bodily functions, including cognitive abilities, mood [73], immune reactions [74,75], motility [73], and active water absorption of the intestine [76,77]. This is the first report that indicates a critical role of the gut microbiota, particularly of commensal Clostridia, in the generation of free catecholamines in the gut lumen and open new horizons in the relationship between human homeostasis and behavior, intestinal physiology and the gut microbiota.

## Crosstalk between Clostridia and gut cells: the immunological point of view

High levels of metabolites produced by Clostridia and their colonization in close proximity to the intestinal mucosa allows us to hypothesize that Clostridia exert a strong influence on the host immune system. Indeed, it has been showed that Clostridia can promote the development of αβ T cell receptor intraepithelial lymphocytes (IEL) and immunoglobulin A (IgA)-producing cells in the large intestine [78]. IEL, IgA-producing cells within the lamina propria, and intestinal epithelial cells are key players in determining the nature of the immunological response to antigens or pathogens ingested. Germ free animals show a reduced number, low Thy-1 expression, and low cytolytic activity, of IEL [79,80]. Furthermore, IgA production is rare [81] and macroscopic Peyer’s patches are small and poorly developed in comparison with those in conventionally-housed animals [82]. Umesaki *et al*. assessed that germ free mice inoculated with 46 strains of Clostridia singly isolated from conventional mice showed an increase in the ratio of CD4^-^ CD8^+^ cells to that of CD4^+^ CD8^-^ in αβIEL within the large intestine. Conversely, the number and phenotype of IEL were similar to those in conventionally-housed mice. The number of IgA-producing cells in the colons of mice treated with Clostridia was slightly increased compared to that in germ free mice [78]. Thus, Clostridia appear to be involved in the promotion of immunological development [78] in the large intestine, but not in the small intestine. The same study showed that in the small intestine, these changes were due to the presence of *segmented filamentous bacteria*[78], suggesting the occurrence of compartmentalization of the immunological responses to indigenous bacteria and of Clostridia in exerting their specific role in gut homeostasis. Moreover, commensal Clostridia are able to normalize cecal size when they are associated with germ free mice [83]. How the immune system fundamentally senses Clostridia remains unclear. In this context, it has been suggested that the presence or gradient of SCFAs and secondary bile acids produced by Clostridia may be sensed by epithelial cells and in turn, may be associated with the initiation of immunological signaling [78], due to the cross talk between epithelial and immune cells. For example, IL-7 secreted by epithelial cells can activate IL-7 receptor-bearing IEL on their progenitors [84,85]. Furthermore, IL-6 [86] and transforming growth factor β [87] produced by the epithelia during infection can stimulate the development of Peyer’s patches and IgA production [88].

*Clostridium spp.* belonging to clusters XIV and IV have also been reported to be strong inducers of colonic T regulatory cell (Treg) accumulation [89]. CD4^+^Foxp3^+^ Tregs are the most prominent regulatory cells in the body and are most abundant in the colonic lamina propria [90,91]. Here, their frequency among CD4^+^ T cells is notably higher than in other organs [89], suggesting that the intestinal microbiota may be involved in the accumulation of colonic Tregs. Several reports have determined that intestinal Foxp3^+^ Tregs are markedly affected by the intestinal microbiota [92]. A fraction of intestinal Tregs express T cell receptors that recognize antigens derived from the gut microbiota [93]. It has been established that these colonic Tregs play critical roles in intestinal immune homeostasis, suppressing systemic and mucosal immune activation to control intestinal inflammation, and contributing to maintaining tolerance towards gut microbiota [94,95]. Atarashi *et al*. showed that colonization of germ free mice with a defined mixture of 46 *Clostridium* strains belonging to clusters XIVa and IV induced the accumulation and differentiation of colonic Tregs [89]. *Clostridium spp.* were also able to promote increased expression of IL-10 in Treg [89], expression of matrix metalloproteinases (MMPs), as well as activation of TGF-β [96] and indoleamine 2,3-dioxygenase (IDO) in colonic epithelial cells [89]. Intestinal epithelial cells are crucial for the maintenance of innate and adaptive immune homeostasis in the gut. Moreover, even the colonization with altered Schaedler flora (ASF), which includes *Clostridium clostridioforme*, leads to the accumulation of Tregs within the colon [97]. Consistent with these findings, *F. prausnitzii*, which belongs to *Clostridium* cluster *IV*, increases IL-10 production from peripheral blood mononuclear cells *in vitro*[98]. How Tregs induced by commensal Clostridia can contribute to immune homeostasis in the intestine is an important question to address. Foxp3^+^ cells with TCRs specific for CBir1, a flagellin related to those of *Clostridium* cluster *XIVa*, induce IgA^+^ B cells in the intestine in order to reduce the mucosal uptake of microbiota-derived antigens and prevent systemic T cell activation [99]. Therefore, *Clostridium spp.* can affect the number and function of colonic Tregs, inducing naive CD4^+^ T cells to differentiate into antigen-specific colonic Tregs that are able to enforce immune tolerance towards commensal bacteria. It is interesting to note that even conventional T cells express TCRs specific for commensal antigens, and are potentially colitogenic if not completely suppressed by intestinal Tregs [100]. Notably, elevated levels of *Clostridium* clusters *XIVa* and *IV* in mice leads to resistance to allergy and intestinal inflammation in experimental models [89]. Conversely, the microbiota of individuals with chronic inflammation show lower bacterial diversity and it has been determined that *Clostridium* clusters *IV*, particularly *F. prausnitzii*, and *XIVa* are significantly less abundant in IBD patients compared to healthy subjects [14,98,101]. It is still unknown whether the decrease in Clostridia is a cause or a consequence of chronic inflammation in IBD patients and in autoimmunity, but we can speculate that they are necessary for immune homeostasis, contributing to the suppression of autoimmunity and deleterious inflammation in humans.

## Conclusions

**Figure 1:**
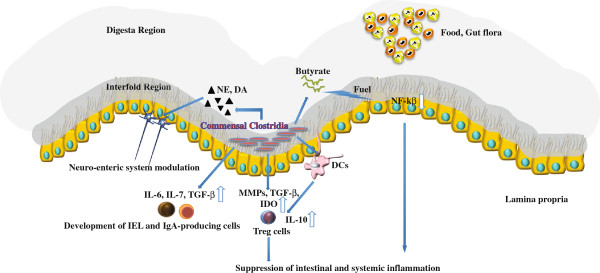
**Peculiar role of commensal *Clostridia *in modulating gut homeostasis.** Establishing a close relationship with gut cells (interfold region), *Clostridia spp.* exert a strong influence on the host immune system. They may be sensed by intestinal epithelial cells and can promote the development of αβ T cell receptor intraepithelial lymphocytes (IEL) and immunoglobulin A (IgA)-producing cells through the induction of IL-6, IL-7 and TGF- β. They are also able to induce colonic T regulatory cell (Treg) accumulation through the activation of Dendritic cells (DCs) and the induction of indoleamine 2,3-dioxygenase (IDO), matrix metalloproteinases (MMPs) and TGF-β in colonic epithelial cells. Furthermore, *Clostridia* play an important role in the metabolic welfare of colonocytes by releasing butyrate as an end-product of fermentation. Butyrate is the preferred energy source for colonocytes, but also inhibits the activation of the transcription factor, NF-kβ, in gut cells. Increased production of IL-10 in Treg cells and decreased expression of NF-kβ lead to a consequent intestinal and systemic anti-inflammatory effect. Finally, *Clostridia*, enriched in β-glucuronidase activity, could be responsible for generating free catecholamines, including Norepinephrine (NE) and Dopamine (DA), from the glucuronide-conjugated biologically inactive form and could be involved in neuro-enteric system modulation.

The present review provides evidence that Clostridia, contributing to a significant portion of indigenous bacteria in the large intestine, are strongly involved in the maintenance of overall gut function. From an experimental point of view, this thesis has been strongly strengthened in a very recent paper [102]. Maurice et al., studying the role of xenobiotics in shaping the physiology and gene expression of the active humane gut microbiota, showed that a distinctive subset of microorganisms, enriched for Clostridia, tends to dominate the active fraction of the gut microbiota [102]. The position of Clostridia, in close relationship with intestinal cells, allows them to participate as crucial factors in modulating physiologic, metabolic and immune processes in the gut (summarized in Figure 1), and appears to be necessary for the welfare of maintaining normal gut immune homeostasis and, on the basis of their influence on the neuroenteric system, of the brain-gut axis. Based on this new information, novel pathogenic hypotheses can be formed that have important translational implications in regard to the prevention and treatment of dysbiosis that can be implicated in many gastrointestinal disorders, including chronic intestinal inflammation, colorectal cancer and irritable bowel syndrome. It will be fascinating to elucidate the underlying mechanisms for xenobiotic resistance and metabolism in the active human gut microbiota in order to provide indications for unexplained patient-to-patient variations in drug efficacy and toxicity. It will also be important to perform detailed mechanistic studies to improve the development of microbial therapies that may modulate the composition of the gut microflora, capitalizing on the key role of commensal Clostridia, with the end goal of promoting gut health.

## Abbreviations

GI: Gastrointestinal; IL: Interleukin; IBD: Inflammatory bowel disease; IBS: Irritable bowel syndrome; TLR: Toll-like receptor; LPS: Lipopolysaccharide; SCFAs: Short chain fatty acids; NF-kB: Nuclear factor kB; DSS: Dextran sodium sulfate; NE: Norepinephrine; DA: Dopamine; GALT: Gut-associated lymphoid tissue; IEL: Intraepithelial lymphocytes; IgA: Immunoglobulin A; Treg: T-regulatory cell; MMPs: Matrix metalloproteinases; IDO: Indoleamine 2,3-dioxygenase; ASF: Altered Schaedler flora.

## Competing interests

The authors declare that they have no competing interests.

## Authors’ contributions

LL did most of the research and writing, and is the guarantor of the article. FS and VP assisted with the editing. AG conceived the original concept for the review, assisted with the research, and performed the editing. All authors have read and approved the final manuscript.
